# Obstructive Sleep Apnea Recognition Based on Multi-Bands Spectral Entropy Analysis of Short-Time Heart Rate Variability

**DOI:** 10.3390/e21080812

**Published:** 2019-08-20

**Authors:** Shiliang Shao, Ting Wang, Chunhe Song, Xingchi Chen, Enuo Cui, Hai Zhao

**Affiliations:** 1 School of computer science and engineering, Northeastern University, Shenyang 110819, China; 2 State Key Laboratory of Robotics, Shenyang Institute of Automation Chinese Academy of Sciences, Shenyang 110016, China; 3 Institutes for Robotics and Intelligent Manufacturing, Chinese Academy of Sciences, Shenyang 110016, China

**Keywords:** heart rate variability, obstruct sleep apnea, power spectrum, Shannon entropy

## Abstract

Obstructive sleep apnea (OSA) syndrome is a common sleep disorder. As an alternative to polysomnography (PSG) for OSA screening, the current automatic OSA detection methods mainly concentrate on feature extraction and classifier selection based on physiological signals. It has been reported that OSA is, along with autonomic nervous system (ANS) dysfunction and heart rate variability (HRV), a useful tool for ANS assessment. Therefore, in this paper, eight novel indices of short-time HRV are extracted for OSA detection, which are based on the proposed multi-bands time-frequency spectrum entropy (MTFSE) method. In the MTFSE, firstly, the power spectrum of HRV is estimated by the Burg–AR model, and the time-frequency spectrum image (TFSI) is obtained. Secondly, according to the physiological significance of HRV, the TFSI is divided into multiple sub-bands according to frequency. Last but not least, by studying the Shannon entropy of different sub-bands and the relationships among them, the eight indices are obtained. In order to validate the performance of MTFSE-based indices, the Physionet Apnea–ECG database and K-nearest neighbor (KNN), support vector machine (SVM), and decision tree (DT) classification methods are used. The SVM classification method gets the highest classification accuracy, its average accuracy is 91.89%, the average sensitivity is 88.01%, and the average specificity is 93.98%. Undeniably, the MTFSE-based indices provide a novel idea for the screening of OSA disease.

## 1. Introduction

Obstructive sleep apnea (OSA) is a common sleep disorder, it is characterized by a partial or complete obstruction of the upper airway during sleep [1]. According to statistics, about 14% of men and 5% of women have obstructive sleep apnea syndrome, and the incidence of the disease is rising among people around the world [2]. The effects of OSA on the autonomic nervous system (ANS) have been extensively studied [3]. Since heart rate variability (HRV) can react to the ANS state in a non-invasive manner, several indices of HRV have been used in OSA recognition research [4,5].

Traditionally, the methods of analyzing HRV focuses on time domain and frequency domain analysis [6,7,8]. On one hand, time domain analysis mainly uses the standard deviation of all continuous RR intervals (SDNN), the square root of the mean of the sum of the squared of differences between continuous R-R intervals (RMSSD), the percentage of differences between continuous R-R intervals that are exceed 50ms (pNN50), and other indexes to evaluate HRV [9]. On the other hand, frequency domain analysis is to decompose HRV into different frequency components, and their relative strength are quantified as power, including very low frequency (VLF), low frequency (LF), high frequency (HF), total power spectrum (TP) [10]. For instance, the time domain and frequency domain indices of 24-h long-time and 15-min short-time HRV in OSA patients were studied in [11], and the results showed that the time domain index SDNN and pNN50 were lower in OSA patients, and the frequency domain index LF/HF was statistically significant. The short-time HRV signal of OSA patients for 5-min was analyzed in [12], it has shown that the micro-awakening index of OSA patients was related to the time domain index SDNN. The micro-awakening index and the apnea hypopnea index (AHI) were relevant to the frequency domain indexes LF/HF, pVLF, pHF, nLF, nHF. 

However, both the analysis methods of time domain and frequency domain are linear analysis of HRV signals, where it is difficult to reflect the intrinsic nonlinear characteristics of the HRV signal [13,14]. Therefore, the nonlinear analysis methods of HRV for OSA detection have been widely studied. In [15], a study of HRV in OSA patients overnight was studied by Poincare Plot, and the results showed that SD1 showed a diminishing trend with severity of OSA, however this did not reach statistical significance. In [16], the 5-min short-time HRV signal was analyzed by Detrended Fluctuation Analysis (DFA) to uncover nonlinear characteristics of the physiological process associated with sleep apnea. Moreover, entropy analysis is a representative nonlinear analysis method. The disorderly fluctuation of HRV contains the ordered intrinsic dynamic characteristics in cardiac activity, and entropy measurement methods can measure the ordered degree of cardiac activity [17]. In [18], it showed that the sample entropy approach does not show major improvement for OSA detection, its main achievement was the simplicity of computation. In [19], the 5-min HRV segments of OSA patients were studied, and the fluctuation characteristics of HRV were studied by fuzzy approximate entropy. The 10-min HRV signal segments were analyzed by multi-scale entropy to realize OSA screening [20]. The ability of the Shannon entropy and multi-scale entropy to characterize sleep apnea–hypopnea syndrome in HRV recordings during the entire overnight was assessed in [21]. However, these entropy analysis methods were to study the fluctuation characteristics of signals either in the time domain or in the frequency domain, and the entropy values are all based on one-dimensional sequences. At present, there is no study on OSA screening by measuring the complexity of the time-frequency spectrum graph of HRV.

In this paper, eight novel indices of short-time HRV are proposed for OSA screening. These indices are based on the proposed multi-bands time-frequency spectrum entropy (MTFSE) method in this paper. In the MTFSE, firstly, the power spectrum of HRV is estimated by the Burg–AR model, whereby the time-frequency spectrum image (TFSI) is obtained. Secondly, according to the physiological significance of HRV, the TFSI is divided into multiple bands in the frequency domain, hence, several sub-TFSIs are obtained. Last but not least, eight novel indices are obtained by studying the Shannon entropy of different two-dimensional sub-TFSIs and the relationships among them. In order to evaluate the performance of the eight novel MTFES-based indices for OSA screening, K-nearest neighbor (KNN), support vector machine (SVM), and decision tree (DT) classification methods are applied on the eight novel indices.

The rest of this paper is organized as follows. Section 2 introduces the database and the prepossessing method of the data. Section 3 provides a detailed description of the proposed MTFSE approach. Results and a discussion of the results are presented in Section 4. The concluding remarks are drawn in Section 5.

## 2. Data Description and Preprocessing 

In this section, firstly, the data taken from the Physionet Apnea–ECG database were described. Then, a preprocessing procedure was applied to acquire HRV from the original ECG.

### 2.1. Data Description

The data used in this paper is derived from the PhysioNet database [22], and the detailed description of the database can be queried on the website (https://physionet.org/). In this paper, the Apnea–ECG dataset in the PhysioNet database was used [23]. Apnea–ECG dataset contains 70 recordings, each recording contains a single ECG signal digitized at 100 Hz with 12-bit resolution. And it is labeled every minute, indicating whether there was a sleep apnea in the object at this minute. According to sleep apnea time of each recording, 70 recordings were divided into three categories.

Category A contained a total of 40 recordings from 15 males and 1 female, with an average age of 50 years old (29–63 years old). Recordings were defined as having 100 or more minutes with apnea. Category C included 20 recordings from 6 males and 5 female subjects, with an average age of 33 years old (27–42 years old). Recordings were defined as having fewer than 5-min of apnea. Category B contained 10 recordings from 4 males and 1 female subjects, with an average age of 46 years old (39–53 years old), each recording with 10 to 96 min of apnea. In the study of this paper, a total of 60 records of category A and category C are used as experimental data, detailed description as shown in Table 1.

### 2.2. Preprocessing

**Figure 1:**
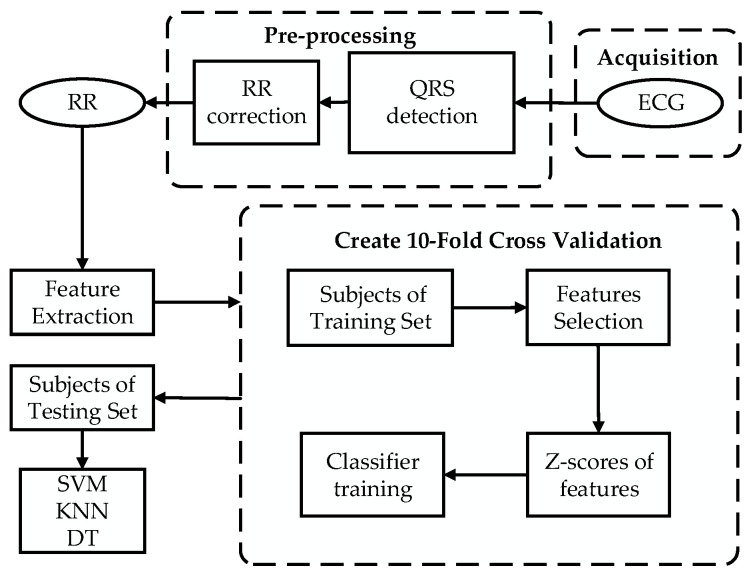
Framework of the obstructive sleep apnea (OSA) screening.

The overall framework of the method proposed in this paper for the screening of OSA patients by short-time HRV signals is shown in Figure 1. In fact, the HRV signal refers to the fluctuation of successive RR interval. Therefore, in order to obtain the HRV series of ECG, the wave group detection of ECG signal was carried out by the QRS wave group (Q wave, R wave, S wave) detection algorithm [24]. However, there will be singularity value in the RR interval obtained by the automatic QRS wave group detection algorithm, so the anomaly point of HRV signal was eliminated by the median filtering algorithm [25]. 

In order to standardize physiological and clinical studies, the guidelines for HRV analysis were proposed by the Task Force of The European Society of Cardiology and The North American Society of Pacing and Electrophysiology [26]. It suggests that short-time recordings of 5-min should be used for the purpose of physiologically stable conditions under frequency-domain analysis. This segment length could balance stationarity and good spectral resolution. Then, according to the method in [19], each HRV signal obtained in class A and class C is divided into several 5-min short-time HRV segments according to the annotation file. Among them, the short HRV segments in class A with at least 1 min of apnea, are defined as the A-OSA group. On the contrary, the segments do not appear apnea in class A, are defined as the A-N group. The short-time HRV signal segments in class C without apnea are defined as the C-N group. The short-time HRV segments of class C with at least 1 min of apnea are not studied in this paper.

## 3. The Proposed MTFSE OSA Detection Method 

**Figure 2:**
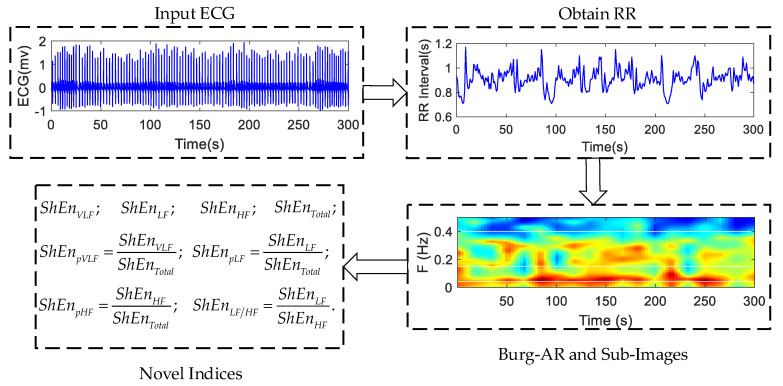
Framework of the proposed multi-bands time-frequency spectrum entropy (MTFSE).

This section provides a detailed description of the proposed approach MTFSE. The framework of the proposed MTFSE is shown in Figure 2. Firstly, the short-time HRV segment is obtained by preprocessing. Secondly, the time-frequency spectrum image is obtained by estimating the power spectrum of the HRV by the Burg–AR model, and, according to the physiological significance of HRV signal, the multi-band segmentation of time-frequency image is carried out in the frequency domain, and three time-frequency sub-images are obtained. Last but not least, the Shannon entropy is calculated separately for the time-frequency image and each sub-image. The Shannon entropy of each sub-image, the ratio of each time-frequency sub-image to time-frequency image, the ratio of low frequency sub-image to high frequency sub-image are the indices for the OSA screening.

### 3.1. AR Power Spectrum Estimation Based on Burg

The power spectrum estimation is an important method for signals in frequency domain analysis. Because the HRV signal is 5-min short-time, so the high resolution of the spectrum is required. Therefore, a modern power spectrum estimation method based on a parametric model is used. The Autoregressive (AR) model has been widely used for power spectrum estimation because of its computational efficiency [27]. Therefore, in this paper, the power spectrum is calculated by the AR model.

In order to ensure the isometric value of the HRV, it is interpolated by three spline interpolation. Then, to ensure resolution of the time domain, the signal after interpolation is re-sampled at a frequency of 4 Hz [28].

The AR model of time series x(t) is described as follows:

(1) $$x\left(t\right)=-\sum_{k=1}^{p}a_{k}x\left(t-k\right)+u\left(t\right)$$

where u(t) is a white noise sequence, and its mean is zero and variance is $\delta^{2}$, where $a_{k}$ is autoregressive coefficient.

As a matter of fact, the AR model is an all-pole model, its transfer function is defined as follows:

(2) $$H\left(z\right)=\frac{1}{1+\sum_{k=1}^{p}a_{k}z^{-k}}$$

Therefore, the power spectrum based on AR model is

(3) $$S_{AR}\left(e^{jw}\right)=\delta^{2}{\left|H\left(e^{j\omega }\right)\right|}^{2}=\frac{\delta^{2}}{{\left[1+\sum_{k=1}^{p}a_{k}e^{-j\omega k}\right]}^{2}}$$

Using the AR model to estimate power spectrum, the parameters of the AR model need to be calculated by the Yule–Walker equation. In this paper, the Burg algorithm is used to solve the Yule–Walker equation [29] because the Burg algorithm can get the required model parameters directly by analyzing the observed data instead of solving the self-correlation function with large computation amounts, and get a more accurate estimate for the short-time signal segment.

The choice of model order is an important step to ensure the accuracy of power spectrum estimation—too low will lead to large mean square error, too high will bring a burden to the calculation. Previous studies have shown that when the sampling frequency is 4 Hz, AR mode can get a high quality power spectrum when the order set to 16 [28]. Therefore, the model order of this paper was uniformly set at 16 order.

### 3.2. Shannon Entropy Indices Based on The Image of Multi-Band Time-Frequency Spectrum 

The time-frequency image of power spectrum of HRV has different texture and distribution characteristics in different frequency bands. Therefore, in this sub-section, the time-frequency image of the power spectrum is segmented into different frequency bands at first, and then the different sub-images are measured by the Shannon entropy.

#### 3.2.1. Multi-Band Segmentation

**Figure 3:**
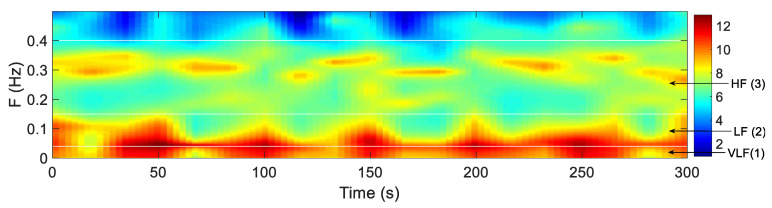
Frequency segmented (white lines) time-frequency spectrum image (TFSI) corresponding to a 5-min of the OSA object’s HRV. Sub-images (1) to (3) represent VLF (0–0.04 Hz), LF (0.04–0.15 Hz), HF (0.15–0.4 Hz) bands.

The power spectrum of HRV is a two-dimensional function of time and frequency, it can be considered as a three-dimensional time-frequency image. As shown in Figure 3, the *x*-axis represents the time, the *y*-axis represents the frequency, and the color value represents the value of the power spectrum. In the image, the white lines represent the dividing line of different sub-images. According to the physiological significance of HRV signal [30], the frequency range of the power spectrum studied in this paper is 0–0.4 Hz, so in this paper, the total time frequency image of power spectrum is defined as TP:0–0.4 Hz. The total time frequency image is divided into three bands according to the following frequency band: Very low frequency (VLF):0–0.04 Hz; low frequency (LF):0.04–0.15 Hz; and high frequency (HF):0.15–0.4 Hz. For these three different bands, the TFSI is divided into three sub-images, and the indices of the HRV signal are extracted by the distribution of the power spectrum in the different sub-image.

#### 3.2.2. Extraction of Shannon Entropy Indices 

Shannon Entropy (ShEn) is related to the amount of information in the signal [31], it can measure the randomness degree of discrete variables. If the probability of random variables taking each value is almost equal, the randomness is stronger, which means that more information is contained and the ShEn value is larger. Conversely, if the probability of a random variable taking a certain number of values is very large, and the probability of taking other values is very small, then the randomness of the variable is weaker, which means that less information is contained and the ShEn value is smaller. Therefore, when the power spectral value of the HRV is more average in the time-frequency image, the ShEn value is smaller. Additionally, the ShEn value is larger when the power spectrum of the HRV is concentrated in a few areas of the time-frequency image (that is, several values of the power spectrum are large and the remaining values are small).

Shannon entropy is defined as follows:

(4) $$ShEn=-\sum_{k}p_{k}\log p_{k}$$

where, k represents the index of the value of the power spectrum. $p_{k}$ represents the probability that the value of the index k appears.

The ShEn of each sub-image is defined as $ShEn_{VLF}$, $ShEn_{LF}$, $ShEn_{HF}$. The ShEn of the total time frequency image is defined as $ShEn_{Total}$. The ShEn ratio of each sub-image ShEn to total image ShEn are defined as $ShEn_{pVLF}=\frac{ShEn_{VLF}}{ShEn_{Total}}$, $ShEn_{pLF}=\frac{ShEn_{LF}}{ShEn_{Total}}$ and $ShEn_{pHF}=\frac{ShEn_{HF}}{ShEn_{Total}}$. The ShEn ration of low frequency sub-image to high frequency sub-image is defined as $ShEn_{LF/HF}=\frac{ShEn_{LF}}{ShEn_{HF}}$. So, in this paper, the index vector *F* used for classification is $F=\left\{ShEn_{VLF},ShEn_{LF},ShEn_{HF},ShEn_{Total},ShEn_{pVLF}，ShEn_{pLF}，ShEn_{pHF}，ShEn_{LF/HF}\right\}$.

## 4. Results and Discussion

Using the proposed indices based on MTFSE method, the HRV signals of A-OSA, A-N and C-N three groups were analyzed. First, the difference analysis of all indices was carried out by t-test. Then, the performance of these statistical significance indices was evaluated by different classifiers. Three kinds of classifiers were selected for comparative analysis, because of the difference in performance of classifiers. These classifiers were K-nearest neighbor (KNN), support vector machine (SVM), and decision tree (DT), and they selected the default parameters in MATLAB2018a.

In order to obtain more reliable and stable results, 10-fold cross-validation was used to divide the objects into testing sets and training sets, and the average value of the 10-fold cross-validation was calculated as the classification results.

Furthermore, the results of the classification were evaluated by three indicators of accuracy (Acc), specificity (Spe), and sensitivity (Sen), which are defined as follows:

Accuracy：$Acc=\frac{TP+TN}{TP+FP+TN+FN}\times 100\%$;

Sensitivity：$Sen=\frac{TP}{TP+FN}\times 100\%$；

Specificity：$Spe=\frac{TN}{FP+TN}\times 100\%$.

where TP is true positive; FP is false positive; FN is false negative; TN is true negative.

In addition, in order to increase the reliability of the results, 300 segments randomly selected from each group for each experiment (A-OSA group: Total 1086 segments, A-N group: Total 660 segments, C-N group: Total 1840 segments). Furthermore, the process was repeated 500 times, at last, the average value of the results of 500 times were used as the final classification results.

### 4.1. Results

The significant difference of indices among the A-OSA, A-N and C-N groups and the performance of the indices are shown in this sub-section.

#### 4.1.1. The Time-Frequency Images of The A-OSA, A-N and C-N Groups

**Figure 4:**
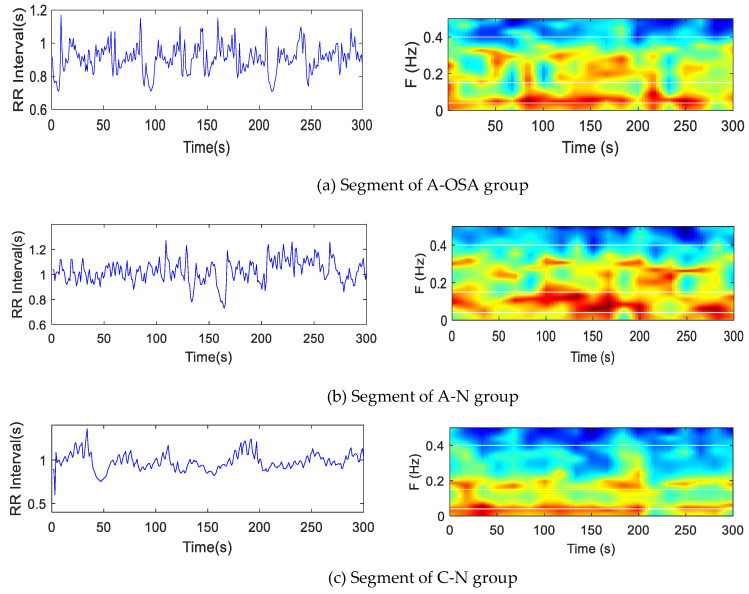
5-min HRV segment (left). 3D color power spectrum of HRV (right). The color represents the power spectrum. (**a**) An apnea segment of OSA group; (**b**) a non-apnea segment of the OSA group; (**c**) a normal segment of the control group.

In order to analyze different disease states, all 5-min HRV segments were divided into A-OSA, A-N, and C-N groups. The HRV segments of objects in A-OSA, A-N, and C-N groups were analyzed by the Burg–AR method. As shown in Figure 4, the overall color of the three sets of data was similar, with three sets of objects having different color distributions on different sub-bands.

#### 4.1.2. The Statistical Significance of Indices Base on The Proposed MTFSE

The A-OSA, A-N, and C-N groups were analyzed by the proposed MTFSE method. In total, 8 indices were obtained based on MTFSE method. Table 2 shows the mean values and stand deviation (SD) of the indices, it also shows the *p*-value of t-test for each of the two groups. The indices $ShEn_{VLF}$, $ShEn_{LF}$, $ShEn_{HF}$, $ShEn_{Total}$, $ShEn_{LF/HF}$, $ShEn_{pVLF}$, $ShEn_{pLF}$, and $ShEn_{pHF}$ were significantly different between the A-OSA and C-N groups (p<0.001). Furthermore, they were also significantly different between the A-OSA and A-N groups (p<0.001). $ShEn_{VLF}$ index was significantly different between the A-N and C-N group (p<0.05). $ShEn_{HF}$ index was significantly different between the A-N and C-N groups (p<0.01), $ShEn_{LF/HF}$, and $ShEn_{pHF}$ were the most statistically significant indices between the A-N and C-N groups (p<0.001).

**Figure 5:**
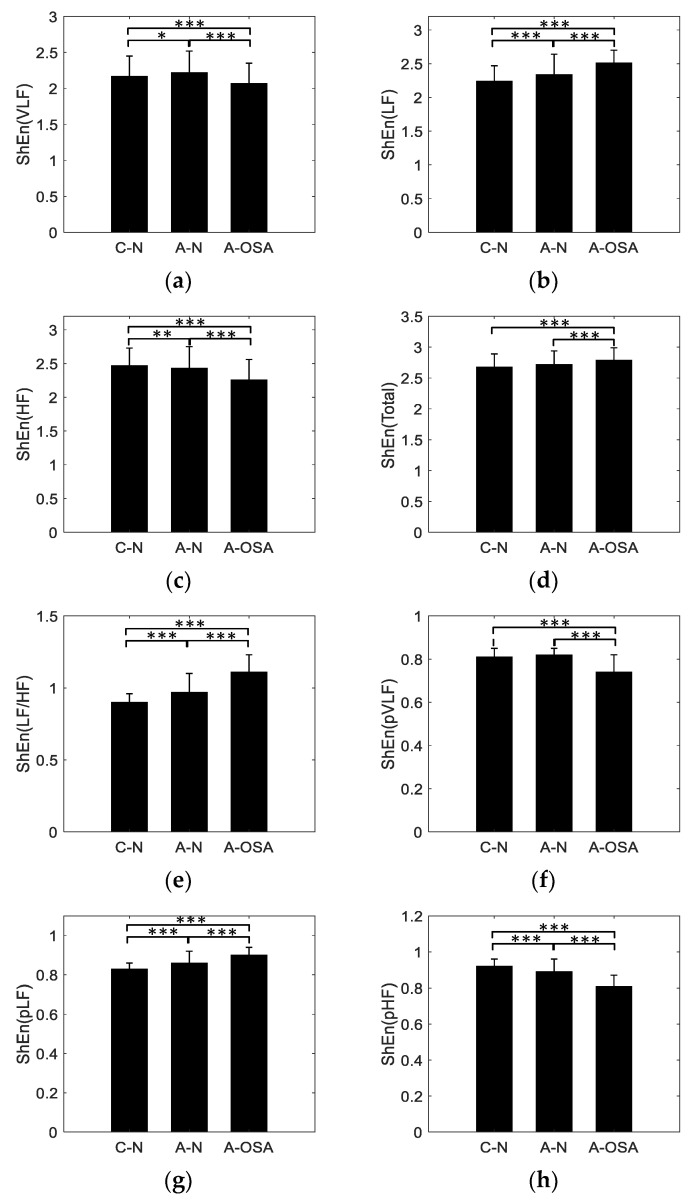
Indices for C-N, A-N and A-OSA groups, (**a**) ShEn (VLF); (**b**) ShEn (LF); (**c**) ShEn (HF); (**d**) ShEn (Total); (**e**) ShEn (LF/HF); (**f**) ShEn (pVLF); (**g**) ShEn (pLF) and (h) ShEn (pHF). *, **, *** represent p<0.05, p<0.01, p<0.001, respectively.

Figure 5 is the mean and SD of the MTFSE-based indices. As can be seen from the Figure 5, the $ShEn_{LF}$, $ShEn_{Total}$, $ShEn_{pLF}$, and $ShEn_{LF/HF}$ indices show an increasing trend among C-N, A-N, and A-OSA groups. The $ShEn_{HF}$ and $ShEn_{pHF}$ indices show a decreasing trend among C-N, A-N, and A-OSA groups. The $ShEn_{VLF}$ and $ShEn_{pVLF}$ indices are the largest in C-N group and in A-OSA group are the smallest.

#### 4.1.3. Performance of the MTFSE-Based Indices

**Figure 6:**
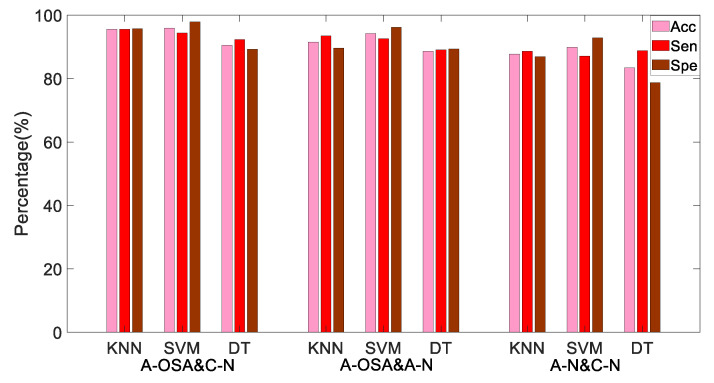
Performance of MTFSE-based indices under different classifiers.

According to the *p*-value of the t-test, $ShEn_{VLF}$, $ShEn_{LF}$, $ShEn_{HF}$, $ShEn_{Total}$, $ShEn_{LF/HF}$, $ShEn_{pVLF}$, $ShEn_{pLF}$, and $ShEn_{pHF}$ are thought to be the most statistically significant indices to discriminate the A-OSA and A-N groups, the A-OSA and C-N groups. $ShEn_{LF}$, $ShEn_{HF}$, $ShEn_{LF/HF}$, $ShEn_{pLF}$, and $ShEn_{pHF}$ are considered as the most statistically significant indices to discriminate the A-N and C-N groups. Through the KNN, SVM, and DT classification methods, the A-OSA and C-N groups, the A-OSA and A-N groups, and the C-N and A-N groups were classified, respectively. The Acc, Sen and Spe of different classification methods between different groups are shown in Table 3 and Figure 6. 

Table 3 and Figure 6 illustrate that for the A-OSA and C-N group, the A-OSA and A-N group, and the A-N and C-N group, SVM shows the highest Acc and Spe, they are 95.94% and 97.92%, 94.20% and 96.22%, 89.87% and 92.86%, respectively. KNN shows higher Sen, they are 95.58%, 93.91%, and 88.62%, respectively. DT has the lowest classification performance.

#### 4.1.4. Performance of OSA Screening

**Figure 7:**
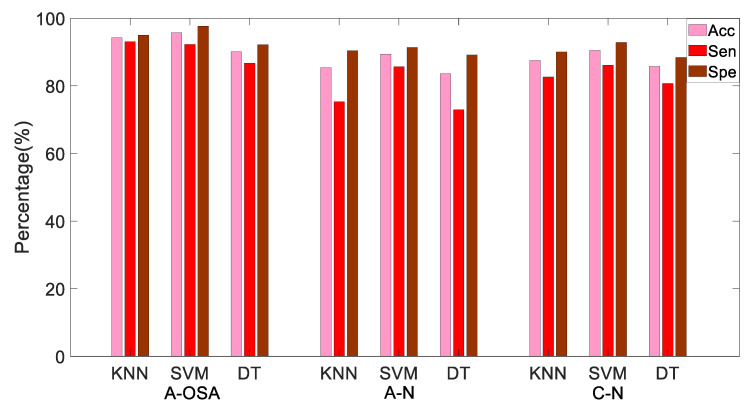
The Acc, Sen and Spe of each classifier including KNN, SVM and DT, respectively.

Moreover, in order to further verify the performance of the proposed MTFSE-based indices, the A-OSA, A-N, and C-N groups were classified at the same time. In this case, the OSA patients can be screened not only in the case of presence of apnea, but also in the case of absence of apnea. The three groups of samples were analyzed by KNN, SVM, and DT, and the Acc, Sen, and Spe are shown in Table 4 and Figure 7. From the results, it can be seen that in the case of SVM, the indices have the highest average Acc, Sen, and Spe, they are 91.89%, 88.01%, and 93.98%, respectively.

**Figure 8:**
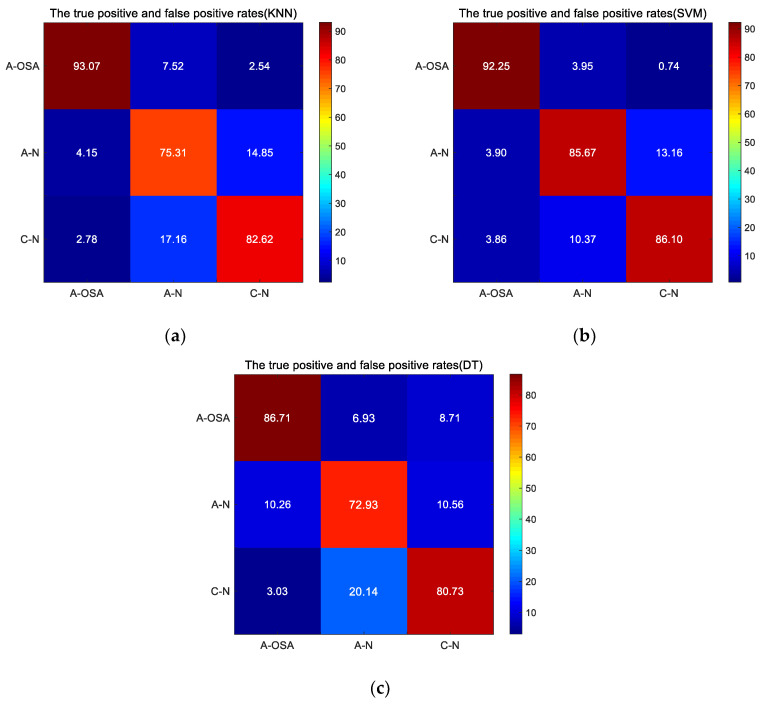
The true positive and false positive rates at different classifier (**a**) KNN; (**b**) SVM; (**c**) DT.

In addition, the true positive and false positive rates at each group are shown in Figure 8 for all classifiers, including KNN, SVM, and DT. As can be seen from the Figure 8, the maximum probability that A-OSA samples are classified into A-OSA class is 93.07% (KNN), and the samples are classified into C-N class with a minimum probability of 2.78% (KNN). The maximum probability that A-N samples are correctly classified into A-N class is 85.57% (SVM), A-N samples are most likely to be mistakenly classified into the C-N class (KNN: 17.15%, SVM: 10.37%, DT: 20.14%). The maximum probability that C-N samples are correctly classified into the C-N class is 86.10% (SVM), the probability that C-N samples are mistakenly classified into A-N class is more than 10% (KNN: 14.85%, SVM: 13.16%, DT: 10.56%) .

## 5. Discussion 

Figure 5 is the mean and SD of the indices based on the MTFSE method. As can be seen from it, the $ShEn_{LF}$, $ShEn_{Total}$, $ShEn_{pLF}$, and $ShEn_{LF/HF}$ indices show an increasing trend among C-N, A-N, and A-OSA groups. The $ShEn_{HF}$ and $ShEn_{pHF}$ indices show a decreasing trend among C-N, A-N, and A-OSA groups. The $ShEn_{VLF}$ and $ShEn_{pVLF}$ indices are the largest in C-N group and in A-OSA group are the smallest. Because existing research shows that LF is thought to be associated with sympathetic activity, variations in the LF spectrum image from HRV reflect changes in the sympathetic nervous system. On the other hand, HF is considered to be related to parasympathetic activity. However, the physiological significance of the VLF is not yet clear, and it has been simply identified with long-period rhythms [32,33,34]. The parasympathetic activity of normal people during nighttime sleep increasing, i.e., in the C-N group, the fluctuation characteristics of the HRV increasing within the HF band, so the texture of HF sub-image of TFSI is complex. In contrast, the sympathetic activity that inhibits HF component decreases, i.e., in the C-N group, the fluctuation characteristics of the HRV decrease with the LF band, so the texture of LF sub-image of TFSI is simple. However, for OSA patients, due to the sympathetic activity being dominant, parasympathetic activity is inhibited. Therefore, compared to normal people, the fluctuation characteristics of the HRV within the HF band decrease and the LF band increase. So the $ShEn_{LF}$ and $ShEn_{pLF}$ indices show an increasing trend among C-N, A-N, and A-OSA groups, on the contrary, $ShEn_{HF}$ and $ShEn_{pHF}$ indices show a decreasing trend among C-N, A-N, and A-OSA groups. Furthermore, $ShEn_{LF/HF}$ index show an increasing trend. Because the $ShEn_{Total}$ represents the Shannon entropy of the total time-frequency image (0-0.4 Hz), the HRV signal of OSA patients will exhibit more complex fluctuating characteristics due to disease. Therefore, $ShEn_{Total}$ shows an increasing trend between the C-N and A-OSA group. As the fluctuation of HRV signals between OSA patients and normal people is mainly reflected in the HF band and LF band, hence, significant differences were shown between the C-N and A-OSA groups, however, there was no significant difference between the C-N and A-N group.

As you know from Table 3 and Figure 6, the highest classification accuracy between the A-OSA and C-N groups is 95.94%(SVM). These two kinds of samples are the easiest to be distinguished, because the respiratory disorders will occur in A-OSA group samples during sleep, sympathetic nervous system activity will increase. The HRV signal will show more disordered fluctuation characteristics, while the normal person's HRV signal presents more regular and orderly characteristics. This difference can be measured by the power spectrum distribution of HRV. The Acc between A-OSA and A-N groups is 94.20% (SVM), it is lower than that between A-OSA and C-N. Because the HRV in OSA patients showed more complex fluctuating characteristics during the apnea, OSA patients have already had potential sympathetic nervous system activity during the non-apnea. The Acc between A-N and C-N groups is 89.87% (SVM), which is lower than that of the first two cases. Both A-N and C-N groups were samples of absence of apnea, and the fluctuation characteristics inside the HRV signal had some similarity, but the potential sympathetic nervous system activity of the A-N group led to the existence of difference between the A-N and C-N groups.

As you know from Table 4 and Figure 7, the average Acc of classifying the three groups (A-OSA, A-N, and C-N) is 91.89%, because when the classification type increases, the possibility of the samples being classified into similar class is increased. As can be seen from Figure 8, the probability that the A-OSA samples will be classified into the A-N group is greater than the probability of being classified into the C-N group because OSA patients have a potential change in HRV during the non-apnea phase. The probability that the A-N samples are classified into C-N group is greater than the probability of being classified into A-OSA, and at the same time, the probability that the C-N samples are classified into A-N group is greater than the probability of being classified into A-OSA. 

The proposed MTFSE method in this paper is compared with the existing method. In [16], the dataset was also divided into three groups, the same as this paper, and the 5-min HRV signal was also analyzed for OSA screening. It achieved a per-segment Acc of 84.76%, Sen 81.45%, Spe 86.82%. In [19], 5-min HRV segments of OSA patients were categorized as the normal segments of OSA group, apneic segments of OSA group, and normal segments of controls group. Additionally, there was OSA screening through its proposed SlTr-fApEn index and the traditional frequency domain index LF/HF. The results for the OAS screening are that Acc is 86.7%, Sen is 82.5% and Spe is 95%. In [21], OSA screening was studied depending on the gender, and the ability of Shannon entropy and multi-scale entropy to characterize HRV for OSA screening was assessed. Its results showed that the Acc for OAS screening for women was 85.2%, and the Acc for OSA screening for men was 77.6%. In this paper, the proposed MTFSE-based indices and SVM obtained higher Acc (91.89%) and Sen (88.01%) compared with [16], [19], and [21] because in [16], the label of segment was based on the central minute. It did not care how many times of apnea occurs. In [19], it only considered the residual signal and not the other valid information. In [21], it only calculated the entropy characteristics of the power spectrum density based on different frequency bands for OSA are screening, it ignored the time information. However, the proposed MTFSE-indices in this paper consider the physiological significance of HRV signals and the combination of time-frequency and nonlinear characteristics is fully utilized. 

The eight novel MTFSE-indices presented in this paper enable the screening of OSA patients. In addition, the advantage of this paper is that it needs only 5-min of an HRV signal to screen OSA patients, and OSA patients can be screened not only by the A-OSA group, but also by the A-N group. However, many of the existing methods require an all-night HRV signal. In addition, there are few studies to distinguish between non-apnea in OSA patients (A-N group) and non-apnea status in normal people (C-N group). However, compared with normal people, OSA patients also have potential characteristics in a non-apnea status. Therefore, this paper studies this, and the proposed novel MTFSE-based indices could distinguish between the A-N group and the C-N group, and these indices get good performance—the Acc, Sen, and Spe are 89.87%, 87.13%, and 92.86%, respectively.

## 6. Conclusions

In this paper, eight novel MTFSE-based indices are proposed for OSA screening, which is realized by analyzing the short-time HRV signal. The indices are extracted by the MTFSE method. In the MTFSE, firstly, the power spectrum of HRV is estimated by the Burg–AR model, then, the TFSI of power spectrum is obtained. Secondly, according to the physiological significance of HRV, the TFSI is divided into multiple sub-bands. Last but not least, by studying the Shannon entropy of different sub-bands TFSIs and the relationships among them, the fluctuation characteristics of HRV signal are measured, and the detection of OSA patients is realized. In order to evaluate the performance of the MTFSE-based indices, the recordings in the Physionet Apnea–ECG database were used, and each HRV segment was classified by KNN, SVM, and DT classification methods. The SVM classification method had the highest classification accuracy for OSA screening, its average accuracy was 91.89%, the average sensitivity was 88.01%, and the average specificity was 93.98%. Undeniably, the MTFSE-based indices proposed in this paper provides a novel idea for the screening of OSA disease. And in the future, the relationship between the severity of the OSA and indices of short-time HRV will be studied.

## Figures and Tables

**Table 1:** A description of the database.

	Normal	OSA
Record (Numbers)	20	40
Sex(men/women)	6/5	15/1
Age(years)	33 ± 5.8	50 ± 6.6
BMI (kg/m2)	21.5 ± 2.4	30.7 ± 4.6
AHI (events/h)	0.2 ± 0.2	37.1 ± 13.4
Duration of apnea (min)	3.3± 4.9	250.5 ± 77.6

OSA: obstructive sleep apnea; BMI: body mass index; AHI: apnea hypopnea index .

**Table 2:** Statistical analysis results of HRV indices under the proposed MTFSE.

Index	C-N (Mean ± SD)	A-N (Mean ± SD)	A-OSA (Mean ± SD)	*p*-Value A-N & A-OSA	*p*-Value C-N & A-OSA	*p*-Value C-N & A-N
$ShEn_{VLF}$	2.17 ± 0.28	2.22 ± 0.30	2.07 ± 0.28	0 ^***^	0 ^***^	0.0498 ^*^
$ShEn_{LF}$	2.24 ± 0.23	2.34 ± 0.30	2.51 ± 0.19	0 ^***^	0 ^***^	0 ^***^
$ShEn_{HF}$	2.47 ± 0.26	2.43 ± 0.32	2.26 ± 0.30	0 ^***^	0 ^***^	0.006 ^**^
$ShEn_{Total}$	2.68 ± 0.21	2.72 ± 0.22	2.79 ± 0.20	0 ^***^	0 ^***^	0.0616
$ShEn_{LF/HF}$	0.90 ± 0.06	0.97 ± 0.13	1.11 ± 0.12	0 ^***^	0 ^***^	0 ^***^
$ShEn_{pVLF}$	0.81 ± 0.04	0.82 ± 0.03	0.74 ± 0.08	0 ^***^	0 ^***^	0.0560
$ShEn_{pLF}$	0.83 ± 0.03	0.86 ± 0.06	0.90 ± 0.04	0 ^***^	0 ^***^	0 ^***^
$ShEn_{pHF}$	0.92 ± 0.04	0.89 ± 0.07	0.81 ± 0.06	0 ^***^	0 ^***^	0 ^***^

*, **, *** represent p<0.05, p<0.01, p<0.001, respectively.

**Table 3:** Performance of MTFSE-based indices under different classifiers.

Object	KNN			SVM			DT		
	Acc (%)	Sen (%)	Spe (%)	Acc (%)	Sen (%)	Spe (%)	Acc (%)	Sen (%)	Spe (%)
A-OSA&C-N	95.54	95.58	95.70	95.94	94.34	97.92	90.54	92.28	89.26
A-OSA&A-N	91.52	93.91	89.58	94.20	92.60	96.22	88.59	89.06	89.35
A-N&C-N	87.71	88.62	86.87	89.87	87.13	92.86	83.38	88.76	78.68

**Table 4:** The Acc, Sen, and Spe of each classifier including KNN, SVM, and DT, respectively.

Object	KNN			SVM			DT		
	Acc (%)	Sen (%)	Spe (%)	Acc (%)	Sen (%)	Spe (%)	Acc (%)	Sen (%)	Spe (%)
A-OSA	94.24	93.07	94.98	95.72	92.25	97.66	90.10	86.71	92.18
A-N	85.37	75.31	90.43	89.42	85.67	91.40	83.59	72.93	89.14
C-N	87.55	82.62	90.05	90.52	86.10	92.88	85.77	80.73	88.46
mean	89.05	83.67	91.82	91.89	88.01	93.98	86.49	80.12	89.93
